# Terephthalaldehyde–Phenolic Resins as a Solid-Phase Extraction System for the Recovery of Rare-Earth Elements

**DOI:** 10.3390/polym14020311

**Published:** 2022-01-13

**Authors:** Ruth Oye Auke, Guilhem Arrachart, Romain Tavernier, Ghislain David, Stéphane Pellet-Rostaing

**Affiliations:** 1 ICSM, Univ Montpellier, CEA, CNRS, ENSCM, 30207 Marcoule, France; ruth.oyeauke@cea.fr (R.O.A.); stephane.pellet-rostaing@cea.fr (S.P.-R.); 2 ICGM, Univ Montpellier, CNRS, ENSCM, 34095 Montpellier, France; romain.tavernier@enscm.fr (R.T.); ghislain.david@enscm.fr (G.D.)

**Keywords:** solid-phase extraction, terephthalaldehyde, ion exchange, rare-earth elements

## Abstract

Rare-earth elements (REEs) are involved in most high technology devices and have become critical for many countries. The progress of processes for the extraction and recovery of REEs is therefore essential. Liquid–solid extraction methods are an attractive alternative to the conventional solvent extraction process used for the separation and/or purification of REEs. For this purpose, a solid-phase extraction system was investigated for the extraction and valorization of REEs. Ion-exchange resins were synthesized involving the condensation of terephthalaldehyde with resorcinol under alkaline conditions. The terephthalaldehyde, which is a non-hazardous aromatic dialdehyde, was used as an alternative to formaldehyde that is toxic and traditionally involved to prepare phenolic ion-exchange resins. The resulting formaldehyde-free resole-type phenolic resins were characterized and their ion-exchange capacity was investigated in regard to the extraction of rare-earth elements. We herein present a promising formaldehyde and phenol-free as a potential candidate for solid–liquid extraction REE with a capacity higher than 50 mg/g and the possibility to back-extract the REEs by a striping step using a 2 M HNO_3_ solution.

## 1. Introduction

Due to their unique properties (electrochemical, luminescent and magnetic properties), rare-earth elements (REEs) have become essential in the development of high technology devices. Indeed, these elements are commonly used for advanced and green technologies such as magnets (hard disks, wind turbines), batteries of hybrid and electric cars, fluorescent lamps, lasers…etc. Given their importance and supply risk, western government agencies have classified these materials as strategic and critical [1,2]. The treatment of industrial or mining wastes or recycling these elements particularly from end-of-life products are the possibilities that are increasingly considered to reduce the stress on the supply of rare earths [3].

Hydrometallurgy processes and more particularly solvent extraction processes are considered as the reference processes for the separation and purification of REEs. However, the utilization of a large volume of solvent over the different stages represents an inherent drawback to this type of process. To limit the use of solvents and problems associated with their usage (human health and environment concerns), solid-phase extraction seems to be a potential alternative. Indeed, it has the advantage of abandoning organic diluents and the problems related to their use such as the volatility, third-phase formation, decomposition of solvents and extractants. Moreover, the implementation of solid-phase extraction technologies is simpler compared to liquid−liquid extraction.

Many ion-exchange resins were developed as solid-phase. Among them, polystyrene cross-linked resins [4,5] and, more specifically, chelating functionalized resins [6], have shown their interest in the recovery of REEs. Other materials such as graphene oxide-based adsorbents with varying degrees of O-donor ligands were also proposed for the adsorption of REEs [7]. Some limitations such as extraction capacity or agglomeration tendency during the adsorption process encourage the investigation towards simple systems such as resole-type phenolic resins.

The resole-type phenolic resins were studied with regard to their ion-exchange properties coming from their phenolic OH groups and/or from active chelating groups [8,9]. A variety of phenolic resins were investigated for the sorption of various heavy metals [10,11,12,13], metalloids [14], lanthanide cations [15,16,17,18,19] and radionuclides [20,21,22,23].

Despite all the different possibilities offered by this type of resin, their preparation involved usually phenol and formaldehyde which are toxic and classified as carcinogenic, mutagenic and reprotoxic (CMR) substances. The replacement of the formaldehyde was recently proposed using several bio-based, non-hazardous aldehydes with phenol [24,25,26]. Among the aldehydes studied, terephthalaldehyde (TPA) with the presence of a second aldehyde in para-position showed the best reactivity [25,26,27]. Additionally, the synthesized phenol–terephthalaldehyde resin displayed a highly crosslinked network which is an interesting property for the purpose of using them in solid–liquid extraction processes.

Alternatives to phenol were also suggested for the preparation of phenolic resins obtained from biobased phenolic compounds [28]. Those thermosetting polymers exhibit high thermal performances in comparison with conventional phenol–formaldehyde resins [29]. Recently it was demonstrated through a comparative study that resorcinol-based polymer bearing ligand exhibited higher lanthanides extraction efficiency than that of phenol and catechol-based polymers [19].

**Scheme 1:**
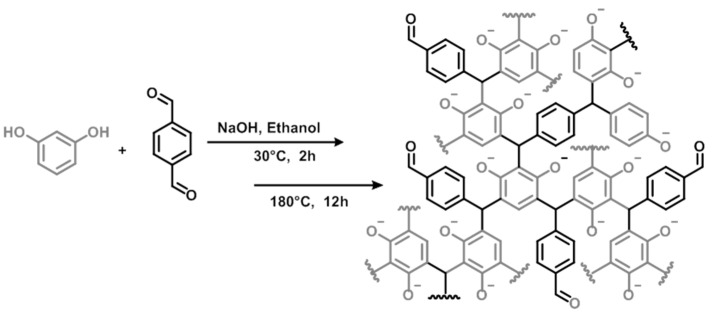
Schematic representation of the synthesized resorcinol–terephthalaldehyde resole resin.

For the above-mentioned reasons, herein we investigate the possibility to use synthesized resorcinol–terephthalaldehyde (RTPA) resole resin for the extraction and recovery of REEs. A schematic representation of the structure of this type of material is illustrated in Scheme 1. Extraction of REEs was performed in a solid–liquid system with the aim to see whether the use of TPA influences the extraction properties of these materials. The use of such solid-phase resin to extract and back-extract REEs was conducted from batch extraction experiments by studying different parameters such as pH effect, as well as metal ion or resins concentration engaged in the sorption experiments. This study further considers the pseudo-first-order and pseudo-second-order rate constants and the kinetics of adsorption of REEs onto the resins. Isotherm and kinetic studies allowed insights into the adsorption mechanisms for such materials.

## 2. Experimental 

### 2.1. Material and Methods

All chemicals were analytically pure (Sigma-Aldrich, Saint Quentin Fallavier, France) and used without further purification. The metal solutions were prepared from nitrate salt of REEs purchased from Acros (Thermo Fisher Scientific, Geel–Belgium) (La(NO_3_)_3_·6H_2_O), Dy(NO_3_)_3_·6H_2_O, Eu(NO_3_)_3_·6H_2_O, Nd(NO_3_)_3_·6H_2_O, Yb(NO_3_)_3_·5H_2_O). 

### 2.2. General Procedure for Resole Preparation

As in a recent work [29], resorcinol (1 equivalent) was introduced into a two-necked flask equipped with a condenser and a magnetic stirrer and dissolved in ethanol (50 wt. %). The obtained solution was stirred at 30–40 °C. TPA (0.4–1.5 equivalent) was added after complete dissolution of the phenolic compound. Then, a 50 wt. % sodium hydroxide solution (0.04 equivalent) was added and the mixture was kept at the temperature as referred earlier. The reaction mixture was periodically analyzed by ^1^H NMR until almost total conversion of TPA. The prepolymer was cooled to room temperature and heated in an oven at 180 °C overnight. Resorcinol—TPA resins were prepared in different molar ratios as summarized in Table 1.

### 2.3. Resin Characterization Techniques

^1^H NMR spectra were recorded in dimethyl sulfoxide-d6 as solvent with a Bruker AC 400 instrument (Brucker, Wissembourg, France) in order to monitor the reactions and to measure conversion of aldehyde function.

The ^13^C solid-state MAS NMR spectra were recorded with a Varian VNMRS 300 solid spectrometer (Varian, Palo Alto, CA, USA) with MAS probe.

IR spectra were recorded with an ATR-FTIR Nicolet 6700 spectrometer (Perkin Elmer, Villebon S/Yvette, France) with a resolution of 4 cm^−1^.

Thermogravimetric analysis (TGA), was performed, under air or nitrogen flow with a heating rate of 10 °C/min from 25 to 950 °C, with a TGA/DSC 2. STARe system (Mettler Toledo, Viroflay, France). After heating in air from 25 to 100 °C at 10 °C/min. the moisture regain was determined by an additional isothermal treatment of 30 min at 100 °C. The water contained in the resin was obtained with the corresponding weight loss.

Differential scanning calorimetry (DSC) was carried out in a DSC-3 F200 Maia (Netzsch, Troarn, France). The atmosphere was dry nitrogen at a flow rate of 50 mL/min with a heating rate of 5 °C/min from −20 °C to 400 °C. 

Scanning electron microscopy was performed using a Quanta 200 ESEM FEG instrument (Thermo Fisher Scientific Electron Microscopy, Lyon France).

### 2.4. Sorption Experiments

#### 2.4.1. Resin Conversion to Na^+^ Form

After curing, the phenolic resin was crushed by ball-milling, and successively washed with 0.1 mol/L HCl/H_2_O and 0.1 mol/L NaOH/H_2_O. Typically, 1 g of resin was shaken with 25 mL of 1 mol/L NaOH for 2 h. The Na^+^ form resin was filtered, washed with water and finally dried in air at 100 °C overnight. The theoretical ion exchange capacity was estimated by equilibrating 0.1 g of resin overnight with 10 mL of 0.1 mol/L NaOH solution. The remaining NaOH was titrated with a 0.1 mol/L HCl solution allowing the determination of the amount of Na^+^ exchanged.

#### 2.4.2. Batch Extraction

Stock solutions of REEs were prepared at the desired acidity in nitric acid from lanthanum (La^3+^), neodymium (Nd^3+^), europium (Eu^3+^), dysprosium (Dy^3+^) and ytterbium (Yb^3+^) starting from their nitrate salts. Extraction experiments were performed using 50 mg of resin equilibrated with 5 mL of metal ion solutions. The mixture was shaken (40–60 rpm) for 24 h to ensure that the equilibrium is considerate, despite that kinetics experiments showed that equilibrium was achieved in 1 h. After filtration over 0.2 µm filter, the supernatant was analyzed by inductively coupled plasma optical emission spectrometry using an ICP-OES SPECTRO ARCOS instrument (SPECTRO AMETEK, Kleve, Germany) with the following wavelengths: La (333.749, 379.478 and 408.672 nm), Eu (381.967, 393.048 and 420.505 nm), Dy (340.780, 353.170 and 394.468) and Yb (222.446, 328.937 and 369.419 nm).

The extraction efficiency E (%) was calculated using the following formula: 

$E=\frac{C_{i\;}-{\;C}_{f}}{C_{i}}\times 100$ where C_i_ is the initial metal ion concentration in aqueous solution (mg/L) and C_f_ is the residual metal ion concentration in aqueous solution after the batch extraction (mg/L).

The equilibrium adsorption capacity Q_e_ (mg/g) is estimated as: $Q_{e}=\left(C_{i}\;-{\;C}_{f}\right)\times \frac{V}{m}$ where V is the volume of the treated solution (mL) and m is the mass of the resin (mg).

The equilibrium of adsorption was described using Langmuir isotherms [30], defined as: $\frac{C_{e}}{Q_{e}}=\frac{1}{Q_{\max}}\times C_{e}+\frac{1}{K\times Q_{\max}}$ where C_e_ is the equilibrium metal ion concentration in liquid phase (mg/L), Q_e_ is the equilibrium metal ion concentration in solid-phase (mg/g), Q_max_ is the maximum metal ion adsorbed/unit mass of adsorbent (mg/g) and K is the Langmuir adsorption equilibrium constant (L/mg). The maximum sorption capacity was estimated from this isotherm when a plateau is obtained [31]. 

Back extraction experiments were carried out using 50 mg of resin equilibrated with 5 mL of a 2 mol/L HNO_3_ overnight. The back-extraction efficiency BE (%) can be obtained as follows $\mathrm{BE}=\frac{Q_{P}-Q_{f}}{Q_{P}}\times 100$ where Q_P_ is the concentration of the metal ion loaded into the polymer and Q_f_ is the residual metal ion concentration in the polymer determined from the residual concentration of metal ions in aqueous solution after back extraction.

#### 2.4.3. Adsorption Kinetic

Kinetic experiments were carried out in the same way as extraction experiments. The residual concentration of metal ions in the supernatant was periodically analyzed by ICP-OES and different experiments were carried out for each time. Kinetics data were fitted using pseudo-first-order and pseudo-second-order kinetic models [32,33].

The pseudo-first-order model suggested by Lagergren based on solid sorption capacity describes the adsorption phenomenon as a diffusion-controlled process suggesting, in the concentration range studied, a formation of monolayer coverage of the adsorbate on the surface of adsorbent [34,35]. This model was used to describe the sorption kinetics [36,37,38]. The pseudo-first-order rate is given as: $\log\left(Q_{e}-Q_{t}\right)=\log Q_{e}-\frac{K_{1}}{2.303}\times t$ where Q_e_ is the equilibrium metal ion concentration in solid-phase (mg/g), Q_t_ is the equilibrium metal ion concentration in solid-phase at time t (mg/g), K_1_ is the pseudo-first-order equilibrium rate constant (min^−1^) and t is the time (min).

The pseudo-second-order model is also based on solid-phase sorption capacity [39,40,41]. The pseudo-second-order rate is given as: $\frac{t}{Q_{t}}=\frac{1}{Q_{e}}\times t+\frac{1}{K_{2}\times Q_{e}^{2}}$ where Q_e_ is the equilibrium metal ion concentration in solid-phase (mg/g), Q_t_ is the equilibrium metal ion concentration in solid-phase at time t (mg/g), K_2_ is the pseudo-first-order equilibrium rate constant (min^−1^) and t is the time (min).

#### 2.4.4. Adsorption Mechanism

Different steps may control the adsorption process. The external particle diffusion can be described as: $\ln\frac{\left(C_{0}-C_{e}\right)}{\left(C_{t}-C_{e}\right)}=\;K\prime t$ where C_0_ is the initial metal ion concentration in aqueous solution (mg/L), C_e_ is the equilibrium metal ion concentration in liquid phase (mg/L), C_t_ is the equilibrium metal ion concentration in liquid phase at time t (mg/L), K′ is the equilibrium constant and t is the time (min) [42]. A straight line demonstrates that the adsorption is characterized by a boundary layer diffusion.

The intra-particle model is expressed by the Weber–Morris equation as:

$Q_{t\;}={\;K}_{i}\times t^{0.5}+C$. where Q_t_ is the equilibrium metal ion concentration in solid-phase at time t (mg/g), K_i_ is the intra-particle diffusion equilibrium constant (mg/g^−1^ min^0.5^), t is the time (min) and C a constant [43,44]. A straight line is evidence that the adsorption is controlled by the intra-particle diffusion only [45]. If there are multi-linear plots, the first one is due to the external adsorption contribution and the slowest step controls the sorption [41].

## 3. Results and Discussion

### 3.1. Resin Synthesis and Characterization

Preliminary investigations were carried out regarding the synthesis of the Resorcinol (R) Terephthalaldehyde (TPA) prepolymer. The molar ratio R: TPA was first optimized. The resins were prepared using one equivalent of resorcinol with a range of TPA from 0.4 to 1.5 and reaction time (t) was optimized to obtain a conversion rate of 95% (Table 1).

**Figure 1:**
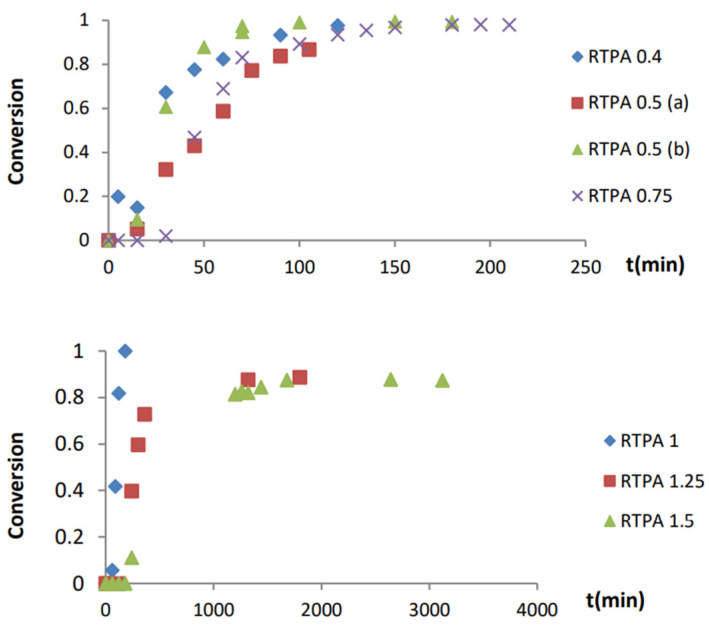
Conversion of TPA into prepolymers vs. time followed by ^1^H NMR experiments.

The prepolymer structure was examined using ^1^H NMR. The methylenol secondary alcohol formed from the addition reaction and leading to a methylene linkage can be observed at 5.6 ppm [29]. A kinetic study allows determining the conversion rate of the reaction with the aldehyde peak at 10.1 ppm, which steadily disappears during the prepolymerization step (Figure 1).

As suggested in a previous study, most of the first aldehyde of TPA was converted during this step [29]. It was observed with the ratio 1:0.5 that 95% of the first aldehyde of TPA were converted within 2 h at 30 °C. This high conversion can be reached in a shorter reaction time by increasing the temperature up to 40 °C. The complete conversion was obtained for the ratios with lower amounts of TPA until 1:1, with a steady increasing reaction time. The prepolymers with the higher amounts of TPA could not exceed 85% conversion even for a longer reaction time. 

The curing behaviors of the prepolymers were evaluated by DSC. Two consecutive reactions were observed with the specific exothermic peaks of thermoset crosslinking reactions (Figure S1 ESI). The total enthalpy ΔH_Total_ of cross-linking values are reported in Table S1 (see ESI). The curves show sigmoidal shapes, corresponding to exothermic peaks, which are typical of thermoset crosslinking. Indeed, the first peak was assigned to the condensation reaction between the first methylenol group of TPA and resorcinol. As reported in the literature, the condensation of the second methylenol group would not occur due to a lack of reactive phenolic position and a lack of resonance forms [29]. Therefore, the second peak was assigned to the degradation of the polymer, which was confirmed from TGA experiments. The obtained values are found about 61 to 113 J/g for the first peak, and from 35 to 116 J/g for the second one. The results highlighted that the reactivity of the formulations is correlated with the amount of TPA since the total enthalpy values decrease when the amount of TPA increases. These results are in good agreement with the observation of the condensation reaction behavior and conversion rate obtained from the NMR analysis.

**Figure 2:**
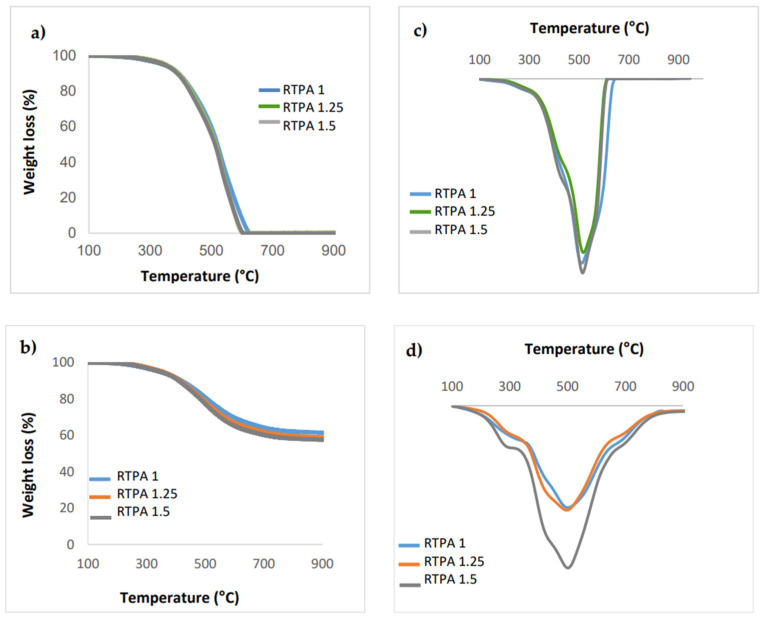
TGA thermograms of fully cured resoles under (**a**) air and (**b**) nitrogen atmosphere; derivative curves under (**c**) air and (**d**) nitrogen atmosphere.

The prepolymers were then engaged in a curing step. The thermal behaviors were investigated using thermogravimetry and differential thermal analysis. The percentage of water in the polymers corresponding to the first slight weight loss is summarized in Table S2 (see ESI). The TGA curves of RTPA 1, RTPA 1.25 and RTPA 1.5 are shown in Figure 2. The weight loss varies from 2% to 5% in agreement with the literature [16,46,47].

Figure 2 displays the superimposed typical resole curves. The weight loss of RTPA 1, RTPA 1.25 and RTPA 1.5 appears in one main stage decomposition proving that the polymer is well cross-linked. Differential curves give a better insight into the phenomena of temperatures. The thermal degradation was highlighted from endothermic peaks in derivatives thermogravimetry curves, which take place over a wide temperature range. As suggested in the DSC analyses, we observed the degradation of the polymer started from 200 °C to 800 °C. However, we observed slight weight losses first before 400 °C, then after 600 °C, and a relevant one between 400 °C and 600 °C, which was attributed to the cross-link degradation involving oxidation of triphenylmethane moiety for TPA. The first weight loss can be assigned to the oxidation of remaining methylenol groups and/or decarbonylation of dangling TPA aldehyde moiety. The last one can be attributed to the ring dehydrogenation forming the amorphous residual char [16,46,47].

The polymers exhibit very high temperatures at 10 wt. % of degradation and char yields reported in Table S3 (see ESI). The curves level off before 900 °C proving the char residues are stable. FT-IR analysis highlighted the presence of carbonyl group in the polymer matrix (Figure S2 ESI). The characteristic band of the aldehyde of the TPA was observed around 1685 cm^−1^ in the resins spectra. Furthermore, the characteristic broad band around 3250 cm^−1^ indicated the presence of O-H bond stretching from the ring hydroxyls, and C-H stretching of the methylene linkage appeared around 2880 cm^−1^.

**Figure 3:**
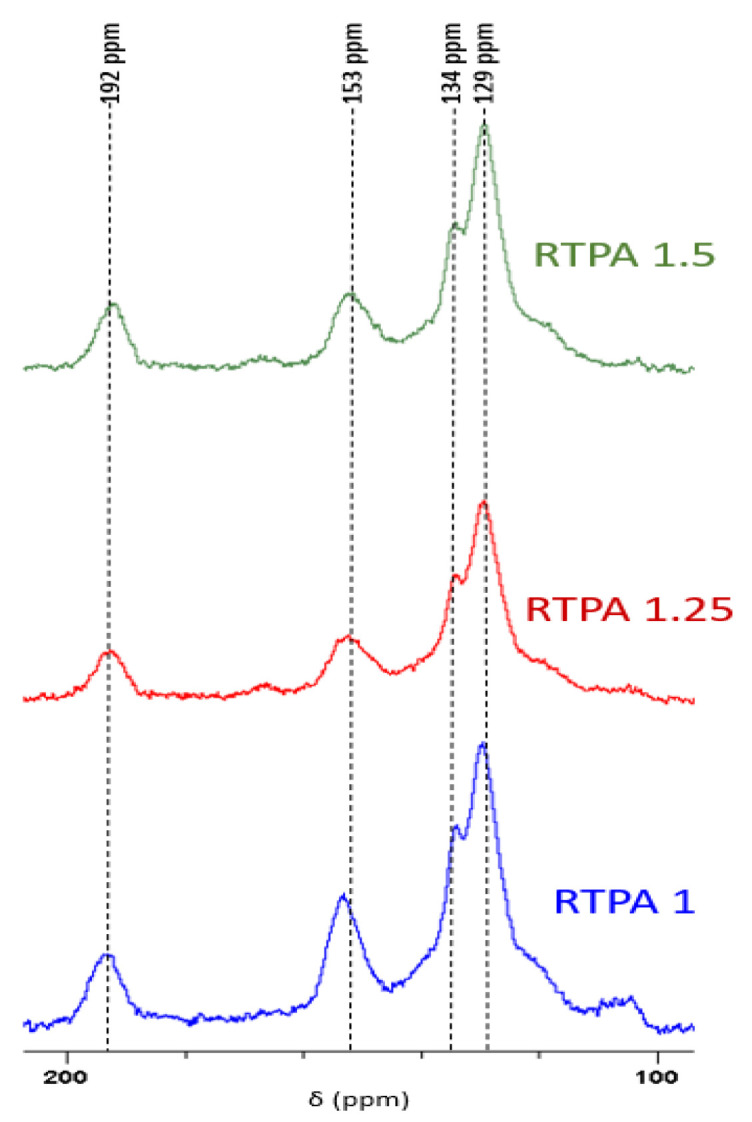
^13^C solid-state NMR spectra of polymers.

Furthermore, after the curing step the ^13^C solid-state NMR shows the displacement of bridging methylene carbons at around 43 ppm and the aromatic carbons and phenolic carbons respectively at 129 ppm and 153 ppm (Figure 3) which can be the consequence of the crosslinking reactions allowing the formation of crosslinked polymers. 

In order to consider the material as sorbent for solid–liquid extraction, the chemical stability of polymers in weak and strong acidic media was investigated. Figure S3 (see ESI) shows an SEM analysis for RTPA 1 cured resole sample before and after chemical treatment in HNO_3_ 2 mol/L for 24 h. No degradation was observed on the polymer surface. Furthermore, IR analyses of cured samples after different chemical treatments were also performed to identify potential changes in the polymer structure. The FT-IR spectra did not reveal any structural change in the polymer matrix (Figure S2 ESI). These results suggest that both morphological and chemical structures are not impacted in the studied conditions.

The theoretical ion exchange capacity of the resins was estimated after conversion to Na^+^ form. The resulting values are presented in Table S4 (see ESI), 88% of Na^+^ from the initial NaOH aqueous solution equilibrated with the resin are exchanged. The theoretical ion-exchange capacity, considering that all the -ONa sites are available in the resin, could be estimated in a range from 400 to 500 mg of lanthanide per g of resin according to the experimental procedures proposed in the literature [16].

The high crosslinked phenolic resins exhibit remarkable thermal and chemical stability. Their potential ion exchange capacity allowed us to study the solid–liquid extraction performance. The resins with higher amounts of aldehyde RTPA 1, RTPA 1.25 and RTPA 1.5, were selected in order to investigate the chelating behaviors of the polymers in the extraction process. 

### 3.2. Sorption Experiments

#### 3.2.1. Extraction and Desorption Experiments

In order to elucidate the chelating behaviors of the polymer, extraction experiments were carried out without any conversion to Na^+^ form. We observed very weak extraction efficiencies, which proves that the carbonyl groups are poorly involved in the extraction process, and the conversion to Na^+^ form enhances the ion-exchange capacity of the polymer. The Na^+^ form resins were used in the different sorption experiments.

**Figure 4:**
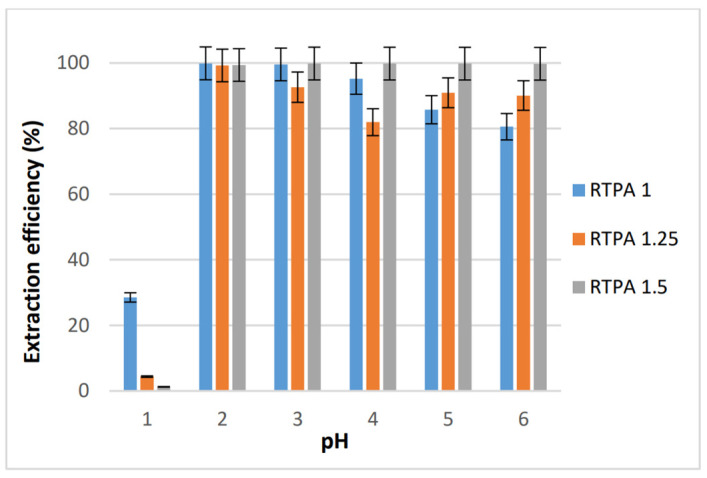
Extraction of Eu by resin RTPA 1. RTPA 1.25 and RTPA 1.5 from feed solution at 50 mg/L as a function of pH.

The cation uptake studies were performed in a pH range of 1–6 in order to examine the efficiency of the resins towards the extraction of Eu^3+^ (Figure 4). Other parameters such as the metal concentration in the aqueous feed solution (50 ppm), solid-mass ratio V/m (V = volume of solution treated and m = mass of polymer used), time and temperature were kept constant. All sorption experiments were performed after a 24 h contact between the resins and different aqueous solutions, despite that equilibrium was achieved in only 1 h, as demonstrated by kinetics experiments.

As illustrated in Figure 4, the resins exhibited high extraction efficiencies for pH values from 2 to 6 (between 80% and 100%). At a pH value of 1, almost no europium was exchanged. The resin is converted back to H^+^ form and this phenomenon is predominant over lanthanide ion exchange during the extraction process. Indeed, the ion-exchange sorption of the cation is controlled by the deprotonation of the phenolic groups. This allows for considering the possibility of an aqueous solution with a pH < 1 in order to perform the back extraction of the metal from the loaded resins. Therefore, in the following experiments, a pH value of 4 was chosen in order to prevent competition between deprotonation and ion exchange, and back extraction experiments were performed with 2 M nitric acid solution knowing that the stability of the resins was demonstrated for this solution.

**Figure 5:**
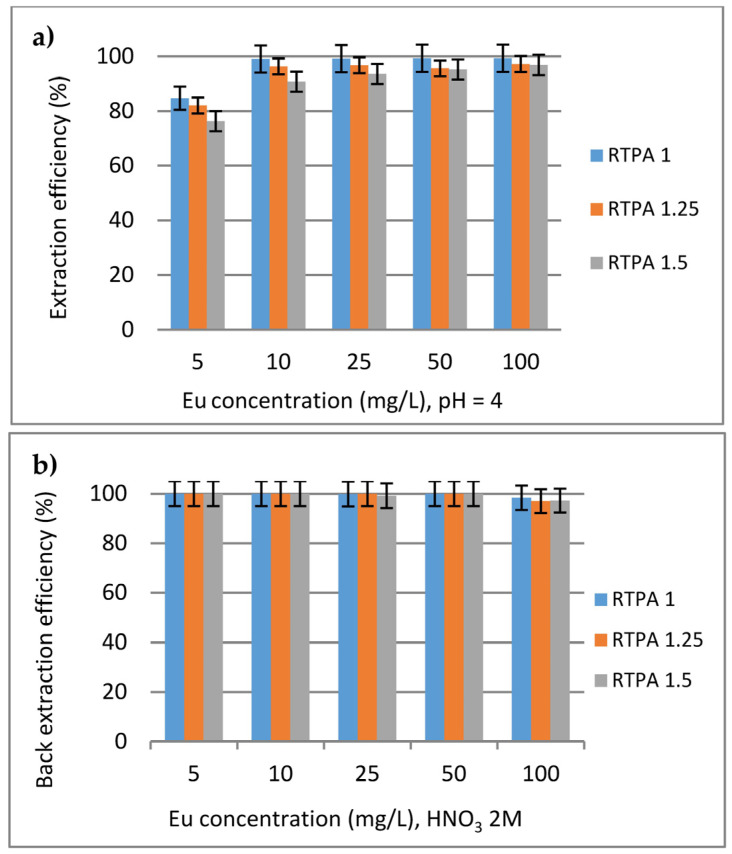
Extraction (**a**) and back extraction (**b**) of Eu by resin RTPA 1, RTPA 1.25 and RTPA 1.5 from cation solutions at various concentrations (pH = 4).

The influence of the metal concentration on the extraction properties of the resins was then studied for Eu^3+^from 5 to 100 mg/L. As illustrated in Figure 5a, very high extraction percentages were reached for all three resins. Additionally, the presence of europium in the polymer after the extraction experiments was monitored by X-EDS analyses (Figure S4 see ESI). Surprisingly, slightly lower extraction efficiency is obtained starting from an acidic solution containing 5 mg/L of Eu^3+^ in comparison to the other concentrations. This can be due to the uncertainties when the cation uptake is performed with a lower initial concentration of metal in the solution.

The back extraction performance was evaluated with HNO_3_ 2M as shown in Figure 5b. It was observed that more than 95% of europium is stripped in all cases. This allows the recovery of the resins which can be then re-engaged in sorption experiments after their conversion to Na^+^ form.

Extraction efficiency was also determined for La, Nd, Dy and Yb as light and heavy REEs. A total of 50 mg of resin was equilibrated with a solution, which contained Dy, La, Eu, Nd, and Yb at a concentration of 50 mg/L for each REE. We observed an almost complete and simultaneous extraction of the lanthanide for all three resins with a slight reduction in the adsorption capacities due to the competition between REEs (Figure S5 see ESI). The desorption experiments show very high desorption efficiencies in each case.

#### 3.2.2. Adsorption Isotherms

**Figure 6:**
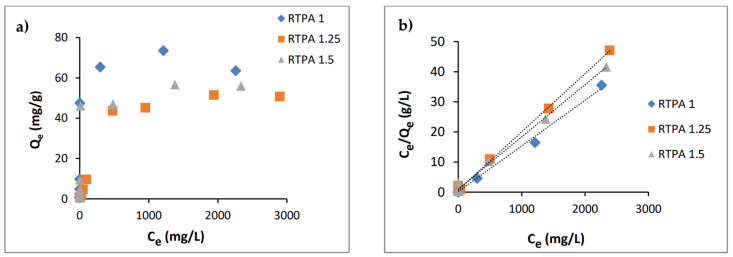
(**a**) Adsorption isotherm for Eu (**b**) Langmuir equilibrium isotherm for RTPA resins.

The isotherm for adsorption of the Eu^3+^ onto the resins at 25 °C was performed from nitric acid solutions (pH = 4) and the initial concentration of Eu^3+^ was varied from 5 to 5250 mg/L, while other parameters were kept constant (Figure S6 see ESI). The results are illustrated in Figure 6. Extraction of Eu^3+^ showed a two-step trend. In the lower concentration range (0–500 mg/L), a rapid increase was observed with a linear correlation between capacity (Q_e_) and the initial concentration of the metal (C_e_) (Figure 6a). This proportionality is no longer observed at the highest concentrations, and a plateau is reached above 500 mg/L. It was observed that at a concentration of 1000 mg/L, the extraction efficiency was reduced by half, suggesting a progressive saturation of the polymer. The resin surface and the adsorption of additional cations are limited at high concentrations. The adsorption behaviors of Eu^3+^ and the surface properties of the resins were investigated using Langmuir’s isothermal adsorption model (Figure 6b). 

The isotherm is well fitted with the Langmuir model of adsorption indicating an isothermal chemisorption mechanism. From the slopes and intercepts of the plots (Table S5 ESI) the Langmuir parameters (Langmuir constant L and maximal sorption capacity Q_max_) were calculated [37]. Thus it could be established that the Q_max_ for Eu^3+^ calculated from the curves were in the range of 50 to 70 mg/g. Therefore, it can be assumed that the surface of the adsorbent is homogeneous in terms of adsorption energy; assuming that all the sites where Eu is adsorbed are similar in energy.

The results indicate that RTPA 1 exhibits the highest adsorption capacities although the different values are roughly similar. Once again, this highlights that the composition of the resins, in the studied condition, has no significant impact on the extraction performance. Moreover, those results are significantly less than theoretical ion-exchange capacities, which were established on the exchange with Na^+^. A plausible explanation for this trend was that the active sites on the resins are not equivalent from the interaction and coordination point of view between Na^+^ and Ln^3+^ and due to the quantity of available adsorption sites.

#### 3.2.3. Adsorption Kinetic Studies

The adsorption capacities to Eu^3+^ were conducted with contact time from 15 to 120 min (Figure S7). Kinetic experiments indicated that the adsorption capacities to Eu^3+^ reach a plateau and therefore the equilibrium was about 1h for the three ratios. RTPA 1 is able to quantitatively extract the metals whereas RTPA 1.25 and RTPA 1.5 have maximum extraction efficiency of 75% and 87%, respectively. 

**Figure 7:**
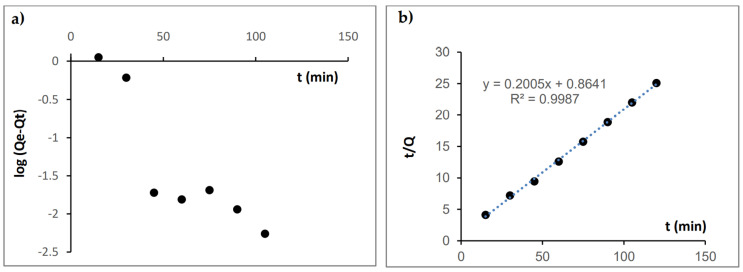
Adsorption kinetics study of Eu by resin RTPA 1 using the (**a**) pseudo-first- and (**b**) pseudo-second-order model.

In an attempt to determine which model is best suited, log (Q_e_ − Q_t_) as a function of time (t) and t/Q versus t were plotted. It appears that the pseudo-first-order model is not suitable since the profile of the curve in Figure 7a is not linear. While a good agreement was found between the experimental data and the pseudo-second-order model which exhibits a high correlation coefficient greater than 0.998 (Figure 7b). Consequently, the result suggested chemical sorption or chemisorption, which can occur by the polar functional group of phenolic resins [40,41].

#### 3.2.4. Adsorption Mechanism Studies

**Figure 8:**
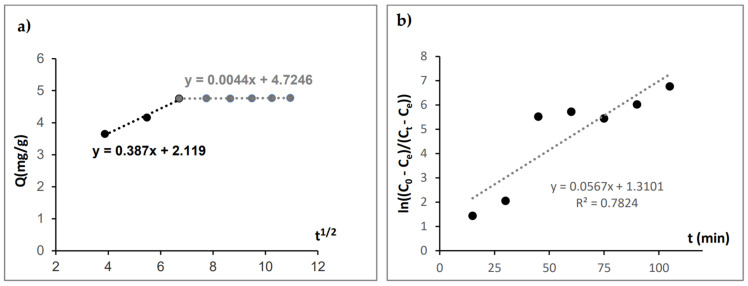
Adsorption mechanism study of Eu by resin RTPA 1 using the (**a**) intra-particle model and (**b**) external model.

The intra-particle model was also compared to the external particle one. Two straight lines were obtained with the first model (Figure 8a). The first one highlighted a boundary layer effect [41]. The very low slope of the second stage is evidence that the intra-particle diffusion does not control the adsorption process, which was confirmed by BET measurement that did not reveal any specific surface area on the polymer. 

The external model showed a boundary layer diffusion (Figure 8b). The non-straight line with a low correlation coefficient obtained through the second model indicates that external diffusion is not the rate-limiting step. Thus, the pseudo-second-order should control the adsorption process [48].

## 4. Conclusions

Resorcinol–terephthalaldehyde resins exhibit a high cross-linked degree and strong chemical stability in various acidic media so that they can be used in solid–liquid extraction processes. At a pH value of 4, 75 up to 100% of metal is removed from initial aqueous solutions containing 5 to 500 ppm of Eu. The present study shows phenolic resins obtained from bio-based and/or non-toxic compounds compete with typical phenol–formaldehyde resins in regards to their extraction properties [16]. It was observed that the Langmuir isotherm equation can be representative of the equilibrium data and suggested a maximum sorption capacity of 70 mg/g for the resin with a molar ratio of 1:1 corresponding to RTPA1. The extraction process follows the pseudo-second-order kinetics and should be controlled by the external mass transfer. Based on these preliminary results, resorcinol–terephthalaldehyde resins seem to be promising formaldehyde and phenol-free candidates for solid–liquid extraction of REE.

## Figures and Tables

**Table 1:** List of phenolic resins.

Resin	Molar Ratio Resorcinol/TPA	T (°C)	Conversion (95%)
RTPA 0.4	1:0.4	30	2 h
RTPA 0.5 (a)	1:0.5	30	2 h
RTPA 0.5 (b)	1:0.5	40	≥1 h
RTPA 0.75	1:0.75	40	2 h
RTPA 1	1:1	40	5 h
RTPA 1.25	1:1.25	40	≤10 h
RTPA 1.5	1:1.5	40	≤50 h

## Supplementary Materials

The following supporting information can be downloaded at: https://www.mdpi.com/article/10.3390/polym14020311/s1, Figure S1: DSC thermograms recorded during the prepolymer RTPA 0.4 curing; Table S1: Total enthalpy of cross-linking values and temperature onset of the exothermic peaks; Table S2: First weight loss corresponding to the percentage of water in the polymer; Table S3: Thermal properties of polymers; Figure S2: FT-IR spectra of RTPA 1 after chemical treatment in HNO_3_; Figure S3: SEM of RTPA 1 before and after contacting with 2 M HNO_3_; Table S4: Theoretical ion exchange capacity of the resins; Figure S4: X-EDS analysis after extraction of Eu 100 mg/L (pH = 4) by resin RTPA 1; Figure S5: Extraction and back extraction of Eu by resin RTPA 1. RTPA 1.25 and RTPA 1.5 from mixed cation solutions with each cation at nominal 50 mg/L concentration (pH = 4); Figure S6: Extraction of Eu by resin RTPA 1. RTPA 1.25 and RTPA 1.5 from cation solutions at various concentrations (pH = 4); Table S5: Langmuir parameters; Figure S7: Extraction kinetics of Eu by resin RTPA 1. RTPA 1.25 and RTPA 1.5.
