# Supplementary material for: Terephthalaldehyde–Phenolic Resins as a Solid-Phase Extraction System for the Recovery of Rare-Earth Elements
Source: Polymers (Basel). 2022 Jan 13;14(2):311. doi: 10.3390/polym14020311 (PMC8780303; doi:10.3390/polym14020311)

Supporting information

Terephthalaldehyde–Phenolic Resins as a Solid-Phase Extraction System for the Recovery of Rare-Earth Elements

Ruth Oye Auke,[1] Guilhem Arrachart,[1,*] Romain Tavernier,[2] Ghislain David[2] and Stéphane Pellet-Rostaing[1]

[1] ICSM, Univ Montpellier, CEA, CNRS, ENSCM, 30207 Marcoule, France
[2] ICGM, Univ Montpellier, CNRS, ENSCM, 34095 Montpellier, France

corresponding author : email: guilhem.arrachart@umontpellier.fr ; phone : +33 4 66 79 15 68
https://orcid.org/0000-0002-5277-3967

Figure S1: DSC thermograms recorded during the prepolymer RTPA 0.4 curing

Table S1: Total enthalpy of cross-linking values and temperature onset of the exothermic peaks

Table S2: First weight loss corresponding to the percentage of water in the polymer

Table S3: Thermal properties of polymers

Figure S2: FT-IR spectra of RTPA 1 after chemical treatment in HNO_3

Figure S3: SEM of RTPA 1 before and after contacting with 2M HNO_3

Table S4: Theoretical ion exchange capacity of the resins

Figure S4: X-EDS analysis after extraction of Eu 100mg/L (pH = 4) by resin RTPA 1

Figure S5: Extraction and back extraction of Eu by resin RTPA 1. RTPA 1.25 and RTPA 1.5 from mixed cation solutions with each cation at nominal 50 mg/L concentration (pH=4)

Figure S6: Extraction of Eu by resin RTPA 1. RTPA 1.25 and RTPA 1.5 from cation solutions at various concentrations (pH = 4)

Table S5: Langmuir parameters

Figure S7: Extraction kinetics of Eu by resin RTPA 1. RTPA 1.25 and RTPA 1.5

Figure S1: DSC thermograms recorded during the prepolymer RTPA 0.4 curing

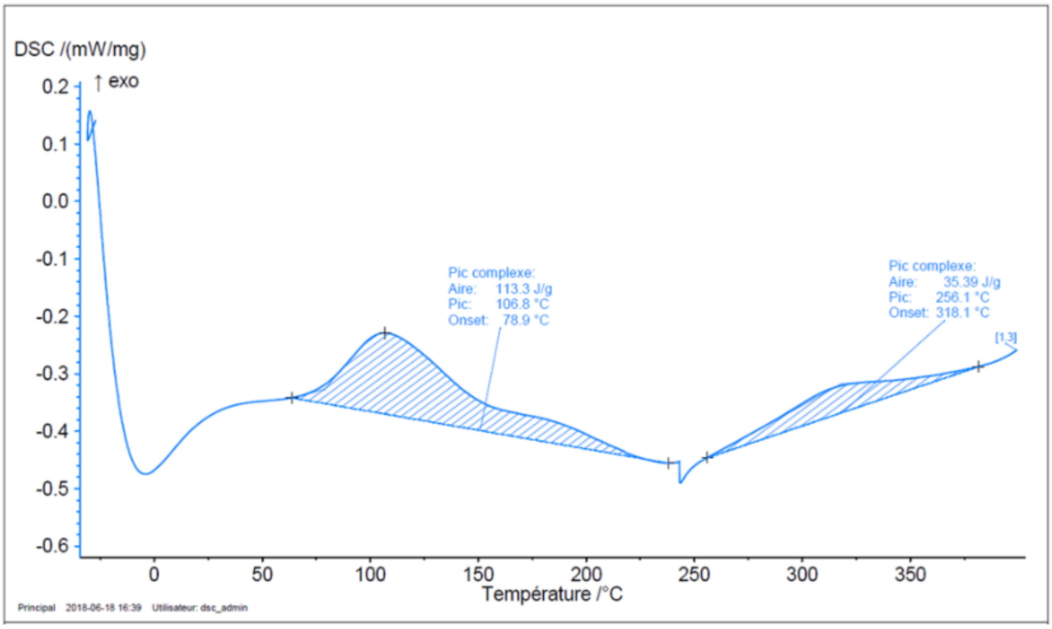

Table S1: Total enthalpy of cross-linking values and temperature onset of the exothermic peaks

Resin	Molar ratio Resorcinol / TPA (eq)	Enthalpie (J/g)		T_{ONSET} (°C)	
		First peak	Second peak	First peak	Second peak
RTPA 0.4	1:0.4	113	35	79	318
RTPA 0.5 (b)	1:0.5	87	10	83	252
RTPA 0.75	1:0.75	71	91	83	272
RTPA 1	1:1	80	96	87	270
RTPA 1.25	1:1.25	61	116	98	251

Table S2: First weight loss corresponding to the percentage of water in the polymer

Resin	Initial weight (mg)	Percentage of resin after isothermal treatment at 100°C	% weight loss
RTPA 1	28.9	95.3301	4.6699
RTPA 1.25	22.8	97.6195	2.3805
RTPA 1.5	26.3	98.0164	1.9836

Table S3: Thermal properties of polymers

Resin	Weight loss after isothermal treatment	$T_{d10\%}$	$T_{d30\%}$	Char yields (%)
RTPA 1	3.4	426	599	61
RTPA 1.25	2.0	420	569	59
RTPA 1.5	1.2	415	555	57

Figure S2: FT-IR spectra of RTPA 1 after chemical treatment in HNO_3

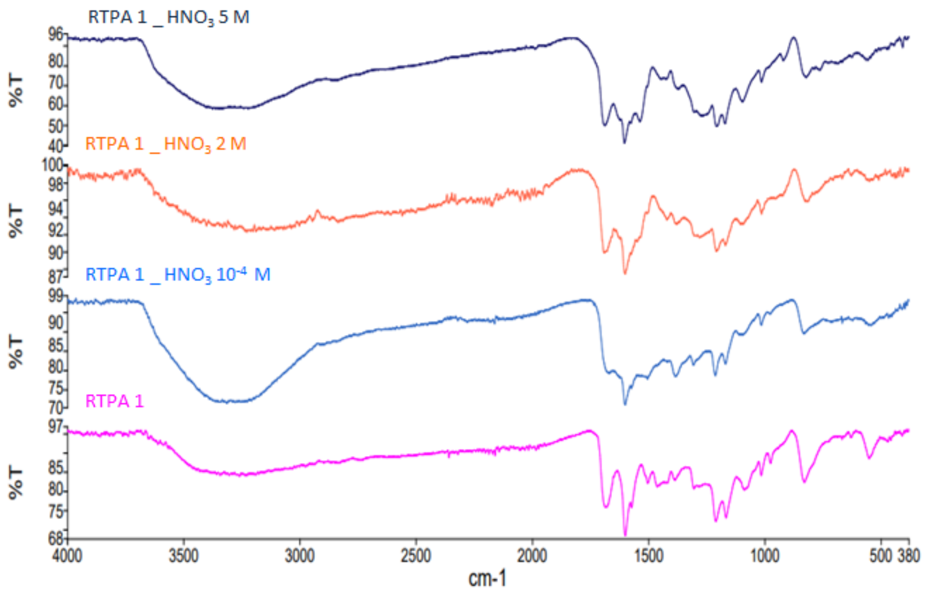

Figure S3: SEM of RTPA 1 before and after contacting with 2M HNO_3

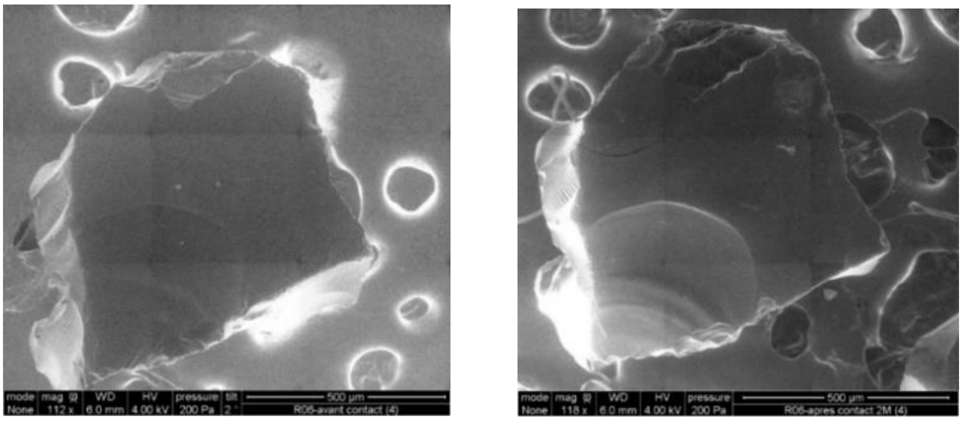

Table S4: Theoretical ion exchange capacity of the resins

Resin	Lanthanide	Ion exchange capacity (Eq of Na^+ / g of resin)	Ion exchange capacity (mmol of Na^+ / g of resin)	Ion exchange capacity (mg of Ln / g of resin)
RTPA 1	Dy	0.293	2.93	476
	Eu			445
	La			407
	Nd			423
	Yb			507
RTPA 1.25	Dy	0.293	2.93	476
	Eu			445
	La			407
	Nd			423
	Yb			507
RTPA 1.5	Dy	0.296	2.96	481
	Eu			450
	La			411
	Nd			427
	Yb			512

Figure S4: X-EDS analysis after extraction of Eu 100mg/L (pH = 4) by resin RTPA 1

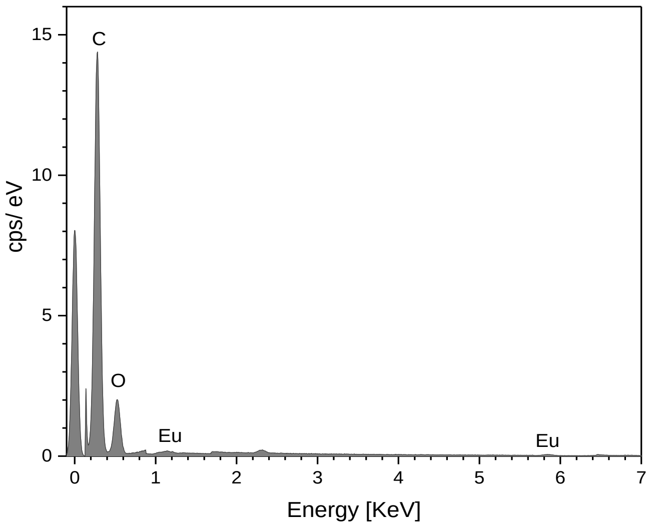

Figure S5: Extraction and back extraction of Eu by resin RTPA 1. RTPA 1.25 and RTPA 1.5 from mixed cation solutions with each cation at nominal 50 mg/L concentration (pH=4)

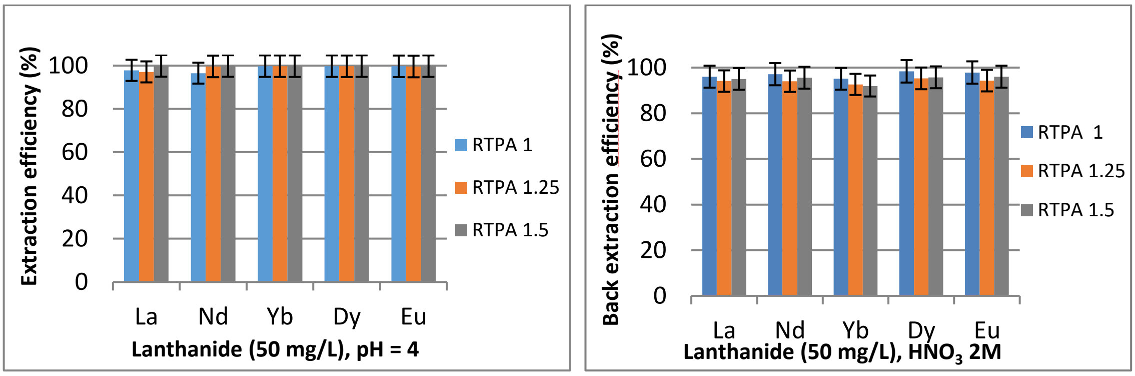

Figure S6: Extraction of Eu by resin RTPA 1. RTPA 1.25 and RTPA 1.5 from cation solutions at various concentrations (pH = 4)

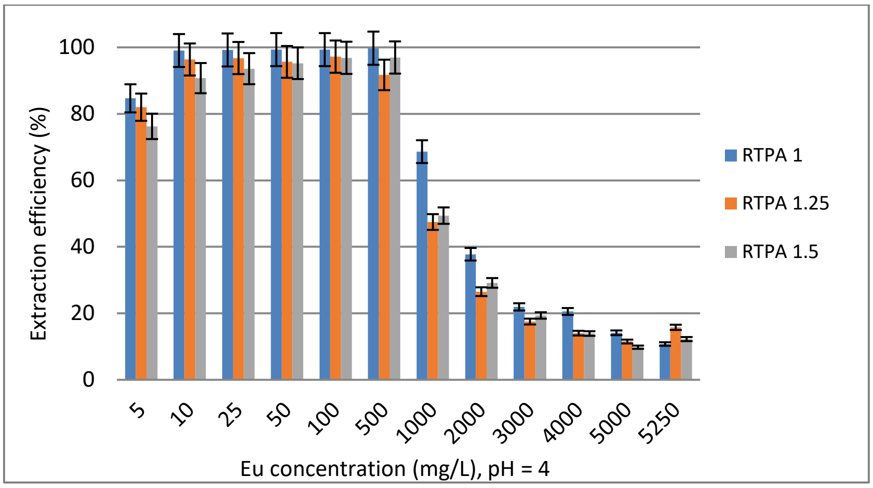

Table S5: Langmuir parameters

Resin	R^2	Q_{max} (mg/g)	K (L/mg)
RTPA 1	0.993	66	$8.94*10^{-2}$
RTPA 1.25	0.9982	52	$2.91*10^{-2}$
RTPA 1.5	0.9957	58	$1.73*10^{-2}$

Figure S7: Extraction kinetics of Eu by resin RTPA 1. RTPA 1.25 and RTPA 1.5.

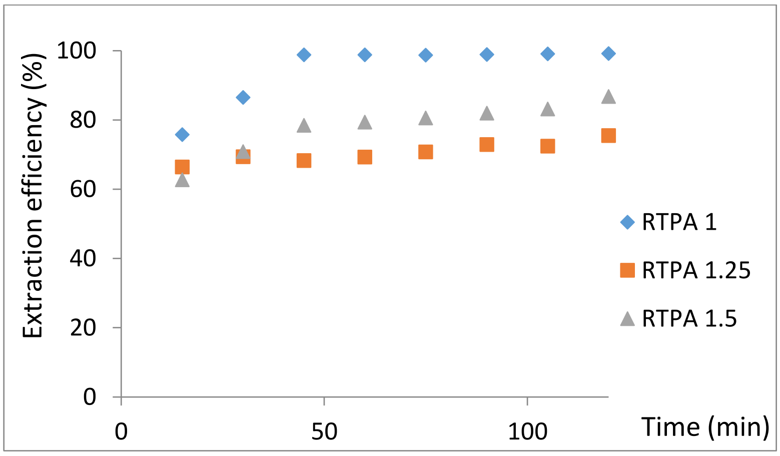

Supplement: Supplementary file 1 [file polymers-14-00311-s001.zip › polymers-1514766-supplementary.pdf]
